# Impact of the COVID-19 pandemic on an online cancer screening training programme for healthcare providers in the public sector in India: learnings from a hub and spoke model perspective

**DOI:** 10.3332/ecancer.2023.1513

**Published:** 2023-02-23

**Authors:** Kavitha Dhanasekaran, Roopa Hariprasad, Mahendra Singh, Sumi Jain, Suzanne Tanya Nethan, Shalini Singh

**Affiliations:** 1 Department of Clinical Oncology, Indian Council of Medical Research – National Institute of Cancer Prevention and Research (ICMR-NICPR), Noida 201301, India; 2 National Programme for Prevention and Control of Cancer, Diabetes, Cardiovascular Diseases and Stroke (NPCDCS), State Government of Chhattisgarh, Raipur, 492101, India; 3 Non-Communicable Diseases, National Health Mission, State Government of Chhattisgarh, 492101, India; 4 School of Preventive Oncology, Patna, 800001 India

**Keywords:** challenges in capacity building during COVID-19, Hub and Spoke model, ECHO model, healthcare providers, cancer screening training

## Abstract

**Introduction:**

This article elicits our experiences and strategic approaches to ensure the sustainability of the online capacity-building programmes for healthcare providers (HCPs) in comprehensive cancer screening through the ‘Hub and Spoke’ model during the coronavirus disease (COVID-19) pandemic.

**Methods:**

During the first wave of COVID-19, training for three cohorts of medical officers (MO) (Batch-A) was ongoing (May–December 2020). The Indian health system abruptly shifted focus towards containing the COVID-19 spread, leading to new challenges in conducting training courses. A new five-step strategic approach for cohort MO-14 (Batch-B) was adopted to spread awareness about the importance of cancer screening and the roles and responsibilities of HCPs in the implementation and conduct of practical sessions in their states in collaboration with their respective state governments. We also adopted social media – *WhatsApp* for official communication.

**Results:**

Enrolling Batch-B following the new strategic approach reduced refusals by 25% and dropouts by 36% compared to Batch-A. Course compliance and completion was a significant 96% in Batch-B.

**Conclusion:**

The COVID-19 pandemic opened a window of opportunity to understand the need for vital changes to improve the quality of our hybrid cancer screening training. Inclusion of the state government in planning and implementing the changes, awareness among HCPs about the importance of training and responsible acceptance of cancer screening, district-wise approach, use of social media in sharing course materials and conducting in-person training in the respective state have demonstrated significant impact on the quality of the training and in scaling-up of cancer screening. Prolonged mentorship, robust Internet connectivity for providers and training on handling gadgets and online video communication would profoundly benefit remote training programmes.

A well-devised backup system is essential for training programmes during unforeseen eventualities such as the COVID pandemic.

## Introduction

The Indian Council of Medical Research-National Institute of Cancer Prevention and Research (ICMR-NICPR) is an India’s premier cancer research institute, with research and prevention as its primary mandate. In addition, it provides training to healthcare providers (HCPs) for implementing nationwide population-based cancer screening programmes.

Since 2015, the institute has adopted Extension for Community Healthcare Outcomes (ECHO), a virtual teaching model to impart its theoretical component of training sessions on cancer prevention using the Zoom platform [1–4]. This ‘Hub and Spoke’ approach has demonstrated its effectiveness in democratising knowledge through the ‘all teach, all learn’ free knowledge policy.

The cancer screening course, which features a standardised curriculum for different cadres of HCPs, has – evolved with time, experience and challenges encountered [1, 5, 6]. We focused on training and strengthening the capabilities of the medical workforce catering to the public sector pan-India as a part of the national cancer screening programme. The Ministry of Health and Family Welfare (MoHFW), Government of India, recognised this course in mid-2019 and recommended that all state governments train their HCPs through this course. ICMR-NICPR is the Indian government-designated training centre for HCPs in cancer screening.

In late 2019, during an ongoing cancer screening course, the world was struck by the coronavirus disease (COVID-19) pandemic. This manuscript’s purpose is to share with the scientific community the impact of the COVID-19 pandemic on conducting online cancer screening training using the ECHO teaching model and coping mechanism adopted to improve the quality of the course.

## Methodology

The NICPR-ECHO online Cancer Screening Training Program (CSTP) is a Health Insurance Portability and Accountability Act (HIPAA)-compliant certification course that uses a hybrid approach, combining online and in-person training using the ECHO teaching model. The CSTP is the only course in India that aligns with national cancer screening guidelines and focuses on capacity building of the HCPs from the public sector. The course has a cadre-specific comprehensive curriculum to train HCPs based on their roles and responsibilities in the national cancer control programme. Capacity building of HCPs (the implementors of cancer control programmes in cancer screening) is an integral part of cancer prevention and a vital pillar of the institute’s mandate.

The CSTP for Medical Officers (MO: CSTP-MO) is a 14-week online course that covers cervical, breast and oral cancer screening and comprises three modules for site-specific cancer screening and management. The curriculum is as shown in Table 1. The sessions consist of weekly 1-hour sessions, starting with the expert’s didactics for 30 minutes on the specific topic in the curriculum, followed by a Q&A session and case presentations by participants. In addition, the participants take quizzes before and after each module and at the end of the course.

Attendance and performance are monitored throughout the course. Participants must achieve a minimum of 70% attendance and 80% aggregate score for the all-module tests to receive a certificate of completion and become eligible for in-person skills training.

In 2019, a systematic approach to training MO serving in the public health sector was initiated in the states of Chhattisgarh, Goa, Tripura, Haryana, Sikkim, Bihar, Andhra Pradesh, Telangana, Tamil Nadu and Karnataka. The course structure, curriculum and methods details are already reported [5–7].

The training programme for three cohorts of MOs (MO11, MO12 and MO13) was ongoing (May 2020–December 2020) during the peak of the COVID-19 pandemic in 2019–2020. The priorities of the health system shifted due to these unforeseen circumstances. As a result, most of our spokes in public service were involved in COVID-19-related healthcare services, and many dropped out of the courses. A survey was sent to dropouts to understand the reasons for dropping out and identify ways to improve participant retention.

As a routine, participants who complete the online course qualify for a 3-day hands-on training programme in cancer screening at our institute in Noida, Uttar Pradesh. However, many qualified participants refused to attend the training expressing their unwillingness to travel, stating reasons such as travel distance, stringent travel restrictions due to the COVID protocol, etc. Therefore, the in-person hands-on training sessions were inevitably postponed until the COVID situation normalised.

During the pandemic, the health system faced immense pressure in managing the colossal unforeseen medical crisis, the loss of normalcy and the sudden surge in COVID cases. It was unable to relieve the HCPs for their role in capacity building in cancer screening. ICMR-NICPR faced numerous challenges in conducting the online course, but these challenges also provided an opportunity to improve the course. With the experience gained in training multiple cohorts, the institute realised that several strategies, changes and enhancements were needed to overcome these challenges, improve course quality and increase participant retention during challenging times such as pandemics.

We also planned to create awareness among the HCPs about the following:

- Cancer burden in their state
- Importance of undergoing formal training in cancer screening
- Nominations from the state government for cancer screening training courses
- Availability of hands-on training in their respective states
- The necessity of performing cancer screening as a routine service

The Non-Communicable Diseases (NCD) division of Chhattisgarh state showed a solid commitment to training its healthcare workforce in cancer screening. Considering their interest and the significant number of nominations, we decided to implement our new course strategy in the cohort from Chhattisgarh.

*Step 1*

We elaborated on the existing situation in a virtual meeting with the Coordinator of NCD, Chhattisgarh (CNC), to reach a consensus on providing awareness to the HCPs about the cancer burden in their state, the need to commence cancer screening at the earliest and the necessity to undergo proper training and hence the nomination to our CSTP.

*Step 2*

The Chhattisgarh state NCD cell made their HCPs aware of our course, its necessity and the benefits of participation in all their major meetings and gatherings. The major points discussed were (a) Cancer screening must be provided as a routine service to beneficiaries and (b) the Importance of undergoing formal training to acquire adequate knowledge and practical skills to perform screening and treatment. The HCPs were also informed that they would all be nominated for the CSTP in a phased manner.

*Step 3*

The NCD cell nominated HCPs serving various health facilities from all over the state in a district-wise approach and communicated this information to the participants through official email and office memorandum. The cell also created an official WhatsApp group for the nominated participants (14th cohort – CSTP-MO14 – Batch-B), including the CNC and the NICPR course convener. The course-related study materials, including PDFs of the expert’s didactics, pre- and post-training quizzes, session reminders, etc., were shared through email and WhatsApp. The participants were allowed to join the WhatsApp group only through invitation. The personal details of the participants/identifiers of the patients are never shared to ensure the privacy of the participants/ patients and the confidentiality of the data.

*Step 4*

The NCD cell, Chhattisgarh, shared the list of nominated participants and their contact details. We then sent invites for course registration to these nominees. To avoid lateral entries from non-registered participants, we adopted the ‘iECHO’ platform – a secure, integrated digital platform that allows registration only via invitation and access to sessions and other services through provided login credentials. This platform additionally features automated attendance tracking that allows monitoring of participants’ retention.

*Step 5*

As cancer screening is a newly assigned responsibility for HCPs, the NCD cell aims to prevent the lack of presentation cases. In order to achieve this goal, the NCD cell connects HCPs with MO who have been trained by ICMR-NICPR and are conducting cancer screenings.

The in-person training for participants is to be conducted in Chhattisgarh state to help them understand the clinical setup and the local context of the programme.

We commenced our training for Batch-B following this five-step approach.

### Statistical analysis

In CSTP-MO12 and 13 (Batch-A) combined, the total number of participants nominated was 276, enrolled 132, case presented 32, total attempted pre-test questionnaire 18 and post-test questionnaire 24, completed online training and qualified for hands-on training 30.

In Batch-B, the total number of participants nominated was 81, enrolled 59, case presented 47, total attempted pre-test questionnaire 53 and post-test questionnaire 45, completed online training and qualified for hands-on training 47.

The data were entered in an MS Excel file and analysed using SPSS version 19 software. The percentages of different parameters were calculated for categorical data.

## Results

Among the 276 participants nominated in Batch-A, refusals to enrol were >50% (144/276). The number of participants who registered was 132, and 53% (70/132) were dropouts. Only 48% (30/62) of the compliant participants completed the course and were eligible for hands-on training. The refusal to enrol and dropouts among the MOs posted in Community Health Centers (CHC) were higher.

**Figure 1.**
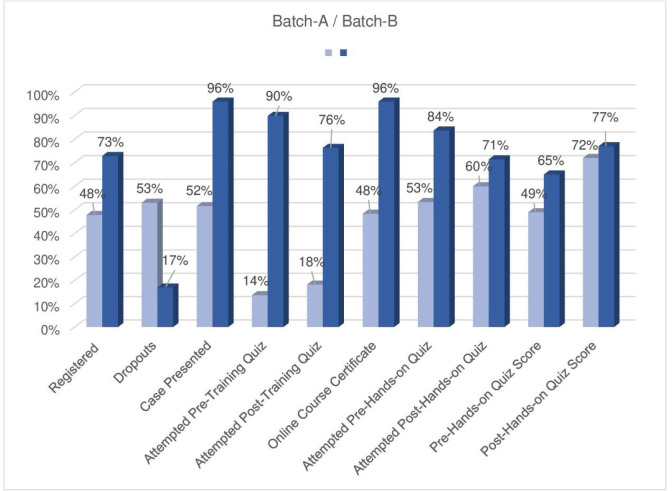
Course compliance details for Batches A and B.

Among the 81 participants nominated in Batch-B, refusals to enrol were 27% (22/81). The number of participants who registered was 59, and 17% (10/59) were dropouts. Only 96% (47/49) of the compliant participants completed the course and were eligible for hands-on training (Figure 1). Details of participants from both batches are shown in Tables 2 and 3. The results of pre- and post-training quizzes for batches A and B are shown in Figure 1.

## Discussion

The study observed the highest refusals (52%) in Batch-A among all the batches trained to date [6]. In our previous batches, the course completion rates among MOs were between 47% and 50%. It was lowest in Batch-A (11%) but with high dropout rates. Participants’ attrition is the major challenge encountered in online courses [8].

Recent studies have reported that the COVID-19 pandemic has improved the uptake of online courses in medical education [9–13]. On the contrary, attrition was highest in our CSTP during the same time. The reason could be that the participants are HCPs – frontline workers in the public health sector. They were involved in sample collection for COVID-19 testing and patient management during the COVID-19 pandemic. The CSTP is only an additional responsibility for HCPs and is a non-mandatory course.

The challenges encountered in conducting CSTP: Internet issues faced by both the hub and the spokes and limited interactions due to the non-use of video during the session were in concordance with challenges faced by courses from other institutes/countries [14, 15].

### Participants’ feedback on the course

**Figure 2.**
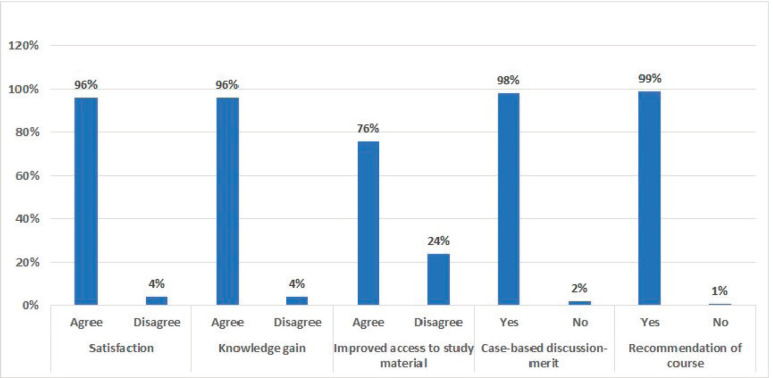
Participants’ feedback on the course.

**Figure 3.**
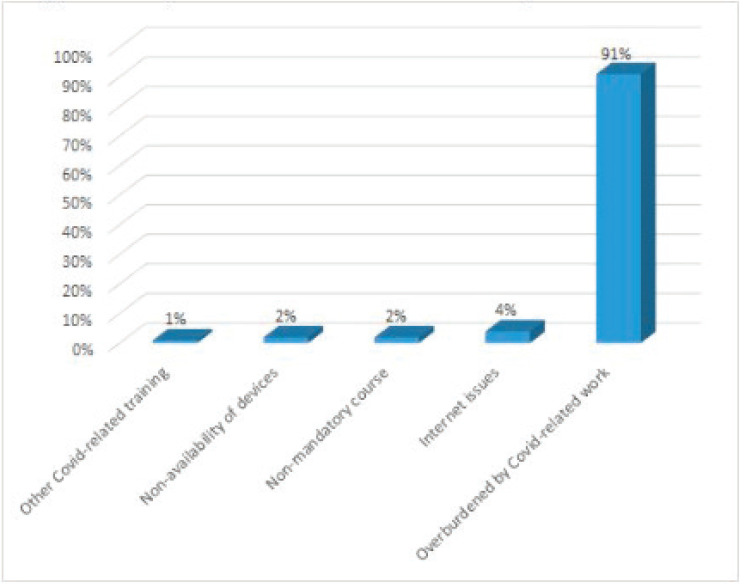
Dropouts’ feedback-reason for dropout.

Maximum participants opined that the course content was helpful and they gained new knowledge through case presentations and discussions. In addition, they were willing to recommend the course to their friends and colleagues (Figure 2). The response rate among the dropouts for the feedback survey was 50%. The reasons for dropouts, as provided by the participants, were: overburdened with other COVID-19-related works, non-availability of devices, other COVID-related training, Internet issues and non-mandatory course (Figure 3).

### Impact of the COVID-19 pandemic on the training programme and the strategies adopted to overcome the challenges

#### The negative impact

##### Participants’ attrition

**Figure 4.**
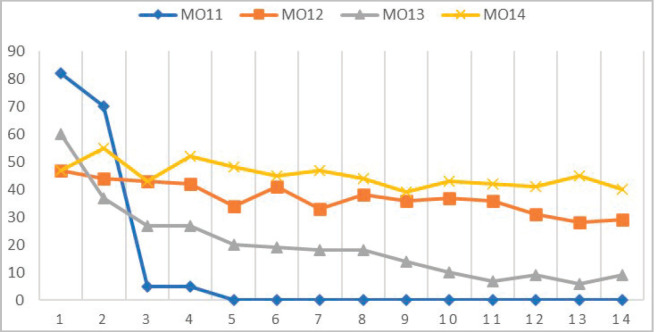
Details of participants’ attrition.

**Figure 5.**
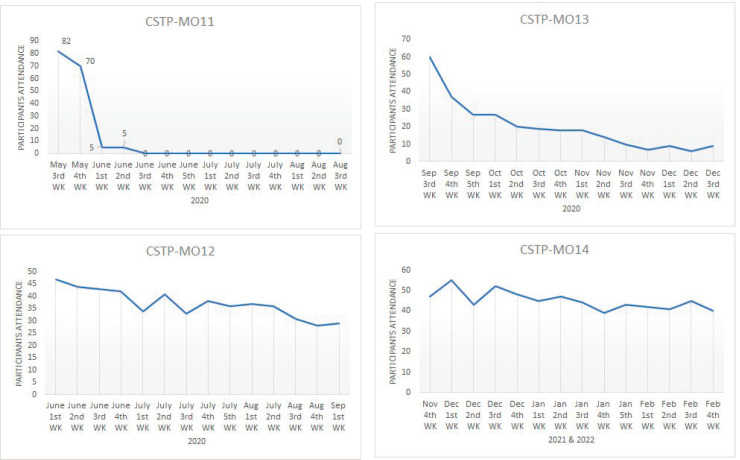
Impact of COVID-19 on participants’ attrition. #The MO11 cohort combined with MO12 from June first week 2020.

HCPs’ priority shifted to the containment of COVID-19 spread. Dropouts were substantial among all three cohorts and highest in MO11, leading to the discontinuation of MO11 as a separate cohort and its combination with MO12 for more efficient utilisation of human resources and to reduce overall stress. Details are given in Figure 4. The frequency of dropouts, the rise in COVID-19 cases and the lockdown had significant correlations [16] (Figure 5).

##### Internet issues

- The work-from-home policy during the lockdown prevented utilising the institute’s robust Internet connectivity and the well-equipped hub infrastructure, which was crucial for the seamless conduct of online sessions.
- Unavailability of high-speed Internet connectivity at all staff residences.
- Generally, increased Internet usage resulted in low connectivity speed that was insufficient for the hub team to share videos and PowerPoint presentations.

We overcame these challenges by utilising high-speed Internet wherever available while others connected through lower-speed mobile data. The team also planned frequent meetings and mock sessions before each live session to ensure successful online sessions. The team also mapped the sequence of who would act as a backup and share the presentations in case of disruptions. All the participants were advised to attend the session with their video switched off and switch it on only when required. However, this was uncomfortable for both the hub and spokes as it restricted interactions.

### Non-availability of subject experts

Due to new additional COVID-related duties, the subject experts were unavailable for their committed sessions. However, we overcame this challenge by utilising the diverse expertise of the hub team, who took over the sessions.

### Non-compliance of the participants to course requirements

Many active attendees needed to comply more with the course requirements – attending pre- and post-module quizzes and presenting cases, drastically decreasing the course completion rate.

### The COVID-19 travel restrictions

The travel restrictions during the pandemic led to the postponement of the hands-on training for Batch-A, which was managed by deferring their hands-on training until the restrictions were lifted. However, the participation rate continued to be considerably lower than expected.

### Refusal of states to participate

When the states were invited to send nominations for a new cohort for training, they refused to participate until the COVID-19 situation normalised.

#### The positive impact

- The learning on the priority shift of the health system during the COVID-19 pandemic and the lull in conducting training courses provided insight into the need to make vital changes to improve participants’ perspectives about the CSTP and our approach towards the participants.
- The lockdowns created ample time to formulate appropriate measures to enforce the necessary changes and improvements to strengthen the quality of the course.
- The COVID-19 pandemic benefited many HCPs by increasing their technological proficiency in utilising devices such as smartphones and laptops and attending online sessions. The arduous challenges managed during this hardship have given new hope and perspective in conducting the online course and realising that dedication, persistence and determination are key elements to success.

#### The new course strategy and its impact

The course completion rate in CSTP was approximately 60% in the pre-COVID era. The new approach and changes made were essential for the HCPs to understand that all health-related programmes should run simultaneously and that cancer screening must be one of their routine activities. In addition, without the new approach, the attrition rate in CSTP could increase, considering the additional responsibilities delegated to the HCPs. Therefore, the changes were imperative for the course’s sustainability and quality.

This initiative resulted in a significant improvement in enrolment among Batch-B’s nominated participants (Figure 1). The participants’ adherence to course requirements, viz. attempting quizzes, presenting cases and attendance – was better (Figure 1). The sessions were more engaging, and continued case discussions and resolving queries in the official WhatsApp group improved rapport with the participants.

Additionally, connecting participants with their ICMR-NICPR-trained contemporaries who were already performing cancer screening in Chhattisgarh proved successful. This strategy motivated participants (Batch-B) to discuss various aspects of the cancer screening programme: counselling, screening, treatment and follow-up, and reduced their hesitance to present cases during the sessions (Figure 1). Finally, engaging the state government in all the stages of the conduct of the CSTP was essential for its success.

The hands-on training for Batch-B in Raipur, Chhattisgarh, provided insight into practical aspects of basic requirements for cancer screening clinics, the supply chain of consumables and referral linkages in their health system. In addition, the Batch-B participants gained substantially higher knowledge after the online training, which further improved after acquiring practical skills.

**Figure 6.**
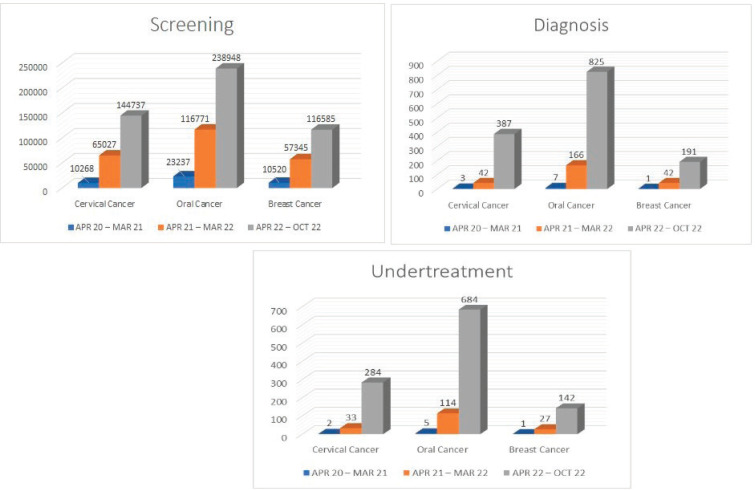
Details of improvement in screening for common cancers.

The new strategic changes in the course made a quantitative impact on the implementation of cancer screening. As a result, the total number of screenings substantially and consistently increased in the Raipur district, where the Batch-B participants were performing screening for common cancers (Figure 6).

A new ‘mentors’ group’ was established, consisting of volunteer-MO from Chhattisgarh state already trained in cancer screening. This mentors’ group created a ripple effect by providing cascaded training to their successors.

#### Strengths and limitations of the manuscript

This manuscript discusses the first-hand experience of the barriers, the facilitators encountered while conducting online CSTP for implementors-frontline workers in population-based cancer screening in India. The improvements in the course compliance metrics forced by the COVID 19 pandemic highlighted inherent and prospective weaknesses in the programme. To address this weakness, the authors emphasise the need for a new strategic approach to improve the course quality and enhance population-based cancer screening. In addition, the authors suggest that periodic evaluations are crucial for the long-term sustainability and impact of the training programmes. However, the precise time duration of each individual’s participation in the session was not captured.

## Conclusion

The COVID-19 pandemic has provided a unique opportunity to assess the limitations and strengths of the current hybrid cancer screening training and make vital changes.

**Five-step strategy:** Including the state government in planning and implementing the changes, increasing awareness among HCPs about the importance of training and responsible acceptance of cancer screening, a district-wise approach and conducting in-person training in the respective state have demonstrated a significant impact on the quality of the training and in scaling up of cancer screening. In addition, continued communication during online courses and prolonged mentorship support also improved the rapport with the participants.

A well-devised backup system is essential for training programmes during unforeseen eventualities such as the COVID pandemic.

Robust Internet connectivity for providers and educating participants on handling gadgets and online video communication would profoundly benefit remote training programmes.

## List of abbreviations

CHC, Community Health Centers; CNC, Coordinator NCD, Chhattisgarh; COVID-19, Coronavirus disease; CSTP, Cancer Screening Training Program; ECHO, Extension for Community Healthcare Outcomes; HCP, Healthcare providers; ICMR-NICPR, The Indian Council of Medical Research-National Institute of Cancer Prevention and Research; MO, Medical officer; MO11, Medical officer cohort 11; MO12, Medical officer cohort 12; MO13, Medical officer cohort 13; MO14, Medical officer cohort 14; MoHFW, Ministry of Health and Family Welfare; NCD, Non-communicable diseases; SBE, Self-breast examination; CBE, Clinical breast examination; FNAC, Fine needle aspiration cytology; HIPPA, Health Insurance Portability and Accountability Act; PDF, Portable Document Format.

## Conflicts of interest

The authors have no relevant financial or non-financial interests to disclose.

## Funding

This work was supported by ECHO India, which provided funding for the project. However, ECHO was not involved in the design, data collection, analysis or interpretation of the study results. It was also not involved in the writing or submission of this manuscript.

## Ethics approval

The study was conducted in accordance with the principles of the Helsinki Declaration. The study was approved by the Institutional Ethics Committee of ICMR-National Institute of Cancer Prevention and Research (No. NICPR/IEC/2020/011).

## Authors’ contributions

Kavitha Dhanasekaran: Conceptualisation, methodology, formal statistical analysis and writing – original draft, review and editing. Roopa Hariprasad: Conceptualisation, review and editing, approval of the final version. Suzanne Nethan and Sumi Jain: Methodology, literature search, review and editing. Shalini Singh and Mahendra Singh: Supervision.

## Figures and Tables

**Table 1.** Curriculum of cancer screening training programme for MO.

No	Title	Learning objectives
1	Introduction to cancer screening and the role of MO in the national cancer screening programme	Burden of cancer in India: prevalence, incidence and mortality of common cancers Risk factors of common cancers What is screening? Types of screening Risks and benefits of screening Introduction to cancer screening in India
**Module 1: Cervical cancer screening**		
2	Methods of cervical cancer screening and their advantage and disadvantages and cervical cancer screening guidelines in India and the rationale behind it	Burden and natural history of cervical cancer Causes, risk factors, signs and symptoms of cervical cancer Anatomy and physiology of transformation zone and squamous columnar junction Screening tests and their pros and cons When to begin screening? For whom? Optimal screening intervals When to stop screening? Impact of HPV vaccination on future screening Operational guidelines of MoHFW, India, for cervical cancer screening
3	Visual inspection with acetic acid (VIA) and its evaluation	Brief anatomy and physiology of transformation zone Principle of VIA Brief step-by-step procedure of VIA with video, including the preparation of 5% acetic acid Interpretations of VIA in brief Briefly discuss the advantages and limitations of VIA
4	Treatment of CIN and screen positives	Types of ablative therapies (cryotherapy and thermal ablation, including videos) Eligibility Contraindications Complications Follow-up Types of excisional methods Eligibility criteria for treatment with excisional methods Follow-up
5	Case scenarios in cervical cancer screening and management	Various cases pertaining to precancerous and cancerous cervical lesions will be discussed here. (VIA +ve, VIA −ve, Screen-and-treat cases, etc.)
	Post-module 1 and pre-module 2 evaluation quiz	To assess the knowledge level of MOs with regard to cervical cancer screening after the training module
**Module 2: Breast cancer screening**		
6	Introduction to breast cancer screening, self-breast examination (SBE) and clinical breast examination (CBE)	Burden of breast cancer Causes of breast cancer Risk factors Symptoms and signs of breast cancer Screening methods Operational guidelines of MoHFW, India, for breast cancer screening Role of follow-up and referral in reducing the burden of cancer
7	Triple assessment for breast cancer screening	Types of diagnostic modalities available for breast cancer screening (triple assessment) Ultrasound: eligibility, advantages and limitations Mammography: eligibility, advantages and limitations MRI: eligibility, advantages and limitations Fine needle aspiration cytology (FNAC)/Biopsy
8	Case scenarios pertaining to breast cancer screening	Various cases pertaining to premalignant and malignant breast lesions will be discussed here.
	Post-module 2 and pre-module 3 evaluation quiz	To assess the knowledge level of MOs about breast cancer screening after the training module. To assess the current knowledge level of MOs about oral cancer screening (and tobacco cessation).
**Module 3: Oral cancer screening and miscellaneous**		
9	Introduction to oral cancer screening and its risk factors	Burden of oral cancer Prevalence, incidence and mortality of oral cancer Causes, signs and symptoms, risk factors Role of tobacco in oral cancer Common sites of oral cancer
10	Common abnormalities of the oral cavity	Screening and diagnostic modalities of oral cancer Efficacy of oral examination Technique of oral examination Operational framework of MoHFW, India, for oral cancer screening
11	Tobacco cessation: counselling and follow-up	Behavioural intervention Pharmacological intervention (with focus on nicotine replacement therapy) Relapse and lapse Tobacco cessation clinic – a brief Target population m-Health – a brief
12	Infection control	Disinfection of equipment Preparation of disinfectants or 0.5% hypochlorite solution Sterilisation of cancer screening equipment and disposal of used items
13	Breaking the news of screen positivity/cancer	How to break the news of screen positive news How to calm down the situation in case of adverse response to the diagnosis
14	Documenting cancer screening results	The various fields in the proforma for cancer screening Who fills it, how often What is the rationale and purpose behind each field

**Table 2.** Facility-wise course compliance details of participants from Batch-A.

Batch-A	*n*	Male	Female	Organisation				
				CHC	PHC	DH	UPHC	CH
Nominated	276	235	41	202	10	46	5	13
Registered	132	106	26	98	6	20	3	5
Dropouts	70	60	10	57	3	8	1	1
Case presented	32	23	9	22	2	4	2	2
Attempted pre-training quiz	18	13	5	15	0	2	1	0
Attempted post-training quiz	24	19	5	15	2	3	2	2
Attempted pre-hands-on quiz	16	23	5	19	6	1	0	2
Attempted post-hands-on quiz	18	41	7	35	2	9	0	2
Course completion certificate received	30	22	8	21	2	3	2	2

CH, Civil Hospital; CHC, Community Health Center; DH, District Hospital; PHC, Primary Health Center; UPHC, Urban Primary Health Center

**Table 3.** Facility-wise course compliance details of participants from Batch-B.

Batch-B	Nos	Male	Female	Organisation						
				CHC	PHC	DH	UPHC	MCH	MH	CH
Nominated	81	63	18	36	33	6	2	1	1	2
Registered	59	44	15	24	27	3	2	1	1	1
Dropouts	10	7	3	9	0	1	0	0	0	0
Case presented	47	35	12	14	27	1	2	1	0	1
Attempted pre-training quiz	53	42	11	19	27	3	2	1	0	1
Attempted post-training quiz	45	34	11	13	26	2	2	1	0	1
Attempted pre-hands-on quiz	41	32	9	11	26	1	1	1	0	1
Attempted post-hands-on quiz	35	26	9	7	24	1	1	1	0	1
Course completion certificate received	47	35	12	14	28	1	2	1	0	1

CH, Civil Hospital; CHC, Community Health Center; DH, District Hospital; MCH, Maternal and Child Health; MH, Medical College Hospital; PHC, Primary Health Center; UPHC, Urban Primary Health Center
